# Nanoparticles transported from aquatic to terrestrial ecosystems via emerging aquatic insects compromise subsidy quality

**DOI:** 10.1038/s41598-019-52096-7

**Published:** 2019-10-30

**Authors:** Mirco Bundschuh, Dominic Englert, Ricki R. Rosenfeldt, Rebecca Bundschuh, Alexander Feckler, Simon Lüderwald, Frank Seitz, Jochen P. Zubrod, Ralf Schulz

**Affiliations:** 1 iES Landau, Institute for Environmental Sciences, University Koblenz-Landau, Fortstraße 7, D-76829 Landau, Germany; 2 Department of Aquatic Sciences and Assessment, Swedish University of Agricultural Sciences, Box 7050, S-75007 Uppsala, Sweden; 3 nEcoTox, An der Neumühle 2, 76855 Annweiler, Germany

**Keywords:** Ecology, Environmental impact

## Abstract

Nanoparticle contaminants enter aquatic ecosystems and are transported along the stream network. Here, we demonstrate a novel pathway for the return of nanoparticles from aquatic to terrestrial ecosystems via cross-boundary subsidies. During their emergence, trichopteran caddisflies carried titanium dioxide and gold nanoparticles into their terrestrial life stages. Moreover, their emergence was delayed by ≤30 days, and their energy reserves were depleted by ≤25%. Based on worst case estimates, it is suggested that terrestrial predators, such as bats feeding on aquatic prey, may ingest up to three orders of magnitude higher gold levels than anticipated for humans. Additionally, terrestrial predator species may suffer from alterations in the temporal availability and nutritional quality of their prey. Considering the substantial transfer of insect biomass to terrestrial ecosystems, nanoparticles may decouple aquatic and terrestrial food webs with important (meta-)ecosystem level consequences.

## Introduction

Over 1,000 products, including sunscreens, textiles, and self-cleaning surfaces, contain engineered nanoparticles or are produced utilizing nanotechnology. As a result, the unintentional introduction of engineered nanoparticles into freshwater environments, mainly via wastewater treatment plant effluents^1^, is increasing^2^ and represents a significant share of the manufacturing production volume^3^. Nanoparticles are transported downstream until their ultimate release into the marine ecosystem. However, the potential for nanoparticle transport from the aquatic environment back to terrestrial ecosystems has been neglected thus far. In this context, the transfer of nanoparticles via merolimnic aquatic insects represents a possible pathway directly into terrestrial food webs. Because nanoparticles are consumed within the insect aquatic life stages, this process likely results in a higher nanoparticle bioavailability for terrestrial species than if they were associated with the soil matrix. Although studies have shown that nanoparticles can be transmitted along the aquatic^4,5^ and terrestrial^6^ food chains, cross-ecosystem dietary exposure has not yet been examined. Additionally, it is unknown whether the temporal availability and quality of these subsidies, namely the emergence pattern and nutritional value (e.g., energy reserves) of aquatic insects, are affected by nanoparticles. Both the trophic transfer of nanoparticles via aquatic subsidies and changes in the availability and quality of this subsidy may be of serious concern for the well-being of terrestrial predators and their offspring, including endangered species of amphibians, lizards, birds, bats and spiders^7–9^. Indeed, a contamination of aquatic systems by chemicals of anthropogenic origin such as metals, pharmaceuticals and others suggest alterations in the aquatic-terrestrial coupling^10–16^ warranting further studies^17^.

**Figure 1:**
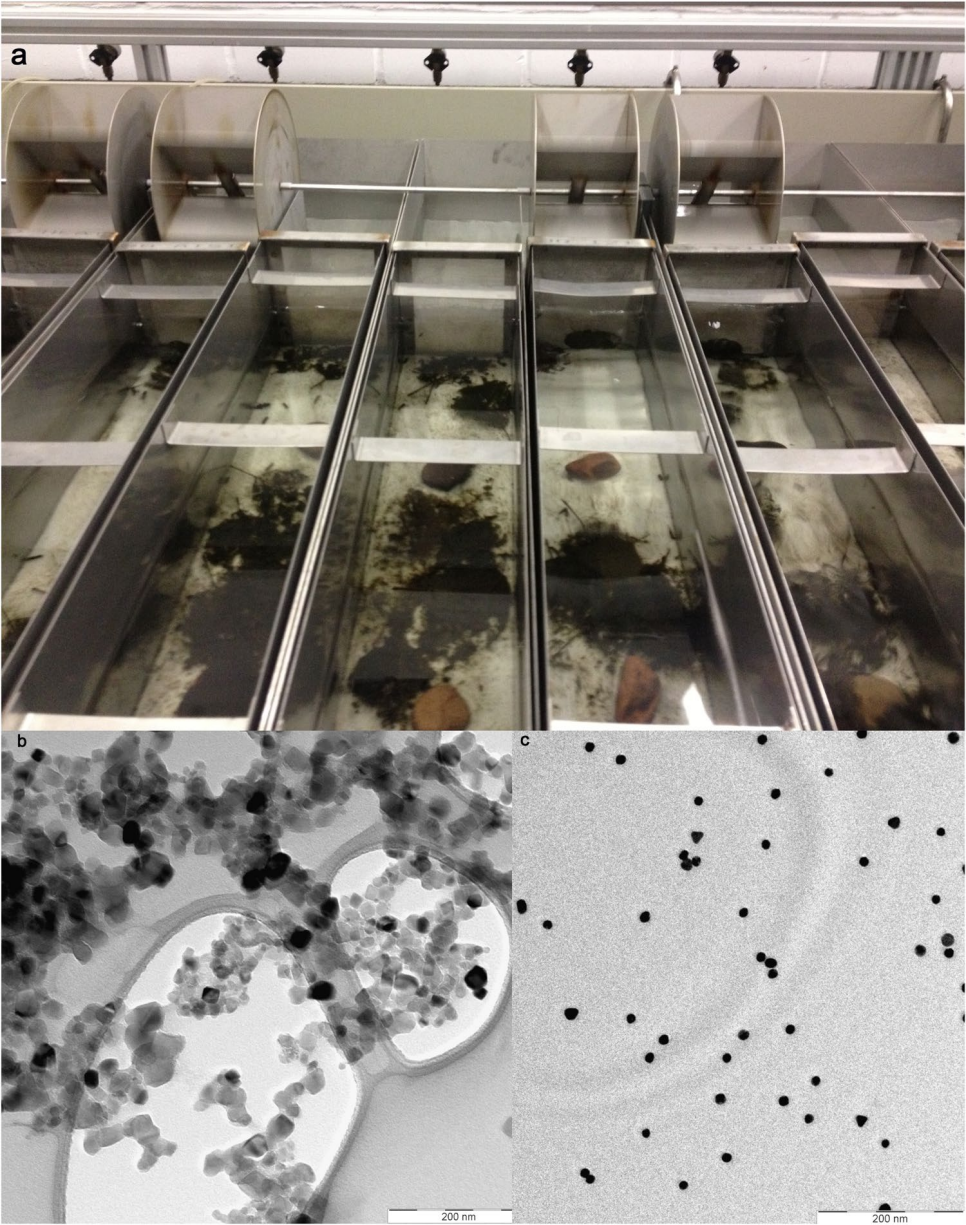
Experimental set-up. (**a**) 24 stainless steel streams (N = 24; volume: 40 L each) simulated freshwater stream habitats in the laboratory. Each stream was equipped with artificial sediment (i.e., glass spheres), leaves and caddisfly larvae. The units were run in flow-through mode, ensuring a constant concentration of nanoparticles over the 140 day duration of the study. (**b**) Transmission electron microscopy (TEM) image of nTiO_2_. (**c**) TEM image of nAu.

To investigate the transfer of nanoparticles from an aquatic to a terrestrial ecosystem, this study employed laboratory flow-through stream microcosms (n = 24; Fig. 1a) over a period of 140 days. In this system, the transfer of 62.3-nm titanium dioxide (4 and 400 µg nTiO_2_/L; Fig. 1b) and 15.1-nm gold (6.5 µg nAu/L; Fig. 1c, see also Table S3) nanoparticles via the caddisfly *Chaetopteryx villosa* developing from aquatic larvae into a terrestrial adult stage was investigated. Nanoparticles were assumed to be taken up by *C*. *villosa* from the water phase or from consumed leaf material that adsorb nanoparticles during exposure (Table S1). In parallel, the temporal emergence pattern and the energy reserves (i.e., lipid stores) of this species, which can be found in large quantities in slow flowing streams^18^, were monitored. In addition, the photocatalytic effects of nanoparticles on the responses of *Chaetopteryx* were assessed by exposure to ultraviolet (UV) irradiation at ambient intensities UV-A: 9.7 W/m^2^, UV-B: 0.35 W/m^2 ^^19^, in the presence and absence of 4 or 400 µg nTiO_2_/L^20^.

## Results and Discussion

**Figure 2:**
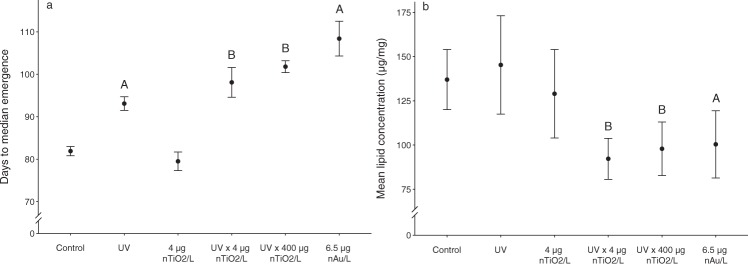
Effects of nanoparticles on caddisflies. Emergence pattern (**a**) and mean lipid concentrations (**b**) in adult caddisflies exposed as larvae for 140 days in stream microcosms to nanoparticles. A and B above the error bars refer to a statistically significant difference relative to the control or the UV treatment, respectively.

Although none of the treatments affected the endpoints overall leaf consumption, survival and emergence rate of caddisfly larvae relative to the controls (Table S2), the temporal emergence pattern (Fig. 2a; Fig. S1), the energy reserves of (Fig. 2b) and the concentration of nanoparticles in the adult organisms (Table 1) deviated considerably among the treatments. The presence of nAu, for example, delayed the emergence (defined as the study duration at which 50% of *Chaetopteryx* individuals have emerged) by up to one month (Fig. 2a). In addition, the energy reserves, measured as total lipid concentration in emerged adults, were reduced by up to 25% (Fig. 2b). UV-irradiation alone delayed emergence by eleven days; in combination with 4 and 400 µg nTiO_2_/L, this effect was significantly extended to 16 and 20 days, respectively, presumably due to the formation of reactive oxygen species^21^. Moreover, lipid concentrations of these adults were significantly decreased (Fig. 2b). Because the lower experimental concentration of nTiO_2_ (i.e., 4 µg/L) is at least seven times less than nTiO_2_ concentrations measured in a recreational lake in central Europe and wastewater treatment plant effluents^22,23^, the delay in emergence caused by the combination of nTiO_2_ and ambient UV-irradiation can (despite the fact that the study was performed under controlled environmental conditions) be considered environmentally relevant with potentially strong implications for aquatic and terrestrial ecosystems. As outlined above, nAu exposure resulted in an even more severe delay in emergence and depletion of energy reserves at a concentration considered to be environmentally safe for Au ions – not nanoparticles, which are usually deemed to be less toxic – based on European regulations^24^. Although the concentration assessed in the present study is an order of magnitude above the nAu concentrations currently predicted for aquatic ecosystems^25^, the data presented here raise concerns for unanticipated effects at the ecosystem level. This concern is underpinned by the substantial effect that shifts in the temporal emergence pattern and the nutritional value (i.e., energy reserves) of emerging adult insects may have on terrestrial predators, such as spiders and adult amphibians, lizards, bats and birds^26^, that may ultimately influence their reproductive success^27,28^. In particular, migratory bird species must fill their energy reserves prior to or following seasonal movements, and changes in the temporal emergence pattern and lipid concentrations of merolimnic prey insects may have serious implications for the birds’ life cycles^8^.

**Table 1:** Concentration of nanoparticles in caddisflies. Concentration of gold and titanium dioxide in adult caddisflies exposed as larvae for 140 days in stream microcosms to nanoparticles.

Treatment	Median Au concentration (ng/mg; range)	Median TiO_2_ concentration (ng/mg; range)
Control	<LOQ	0.43 (0.25–1.09)^a^
UV		
4 µg nTiO_2_/L	NA	0.93 (0.54–18.40)*
UV x 4 µg nTiO_2_/L	NA	
UV x 400 µg nTiO_2_/L	NA	2.68 (0.47–9.44)*
6.5 µg nAu/L	1.51 (<LOQ - 15.33)*	NA

*Statistically significant compared to the control (p < 0.05).

^a^As titanium is among the ten most common elements of the earth crust, the detection of trace levels in organisms are expected even when they were not exposed to nTiO_2_. This was shown, amongst others, by Rosenfeldt *et al*.^43^.

NA = not assessed.

LOQ = absolute limit of quantification (3.6 ng for Au).

**Figure 3:**
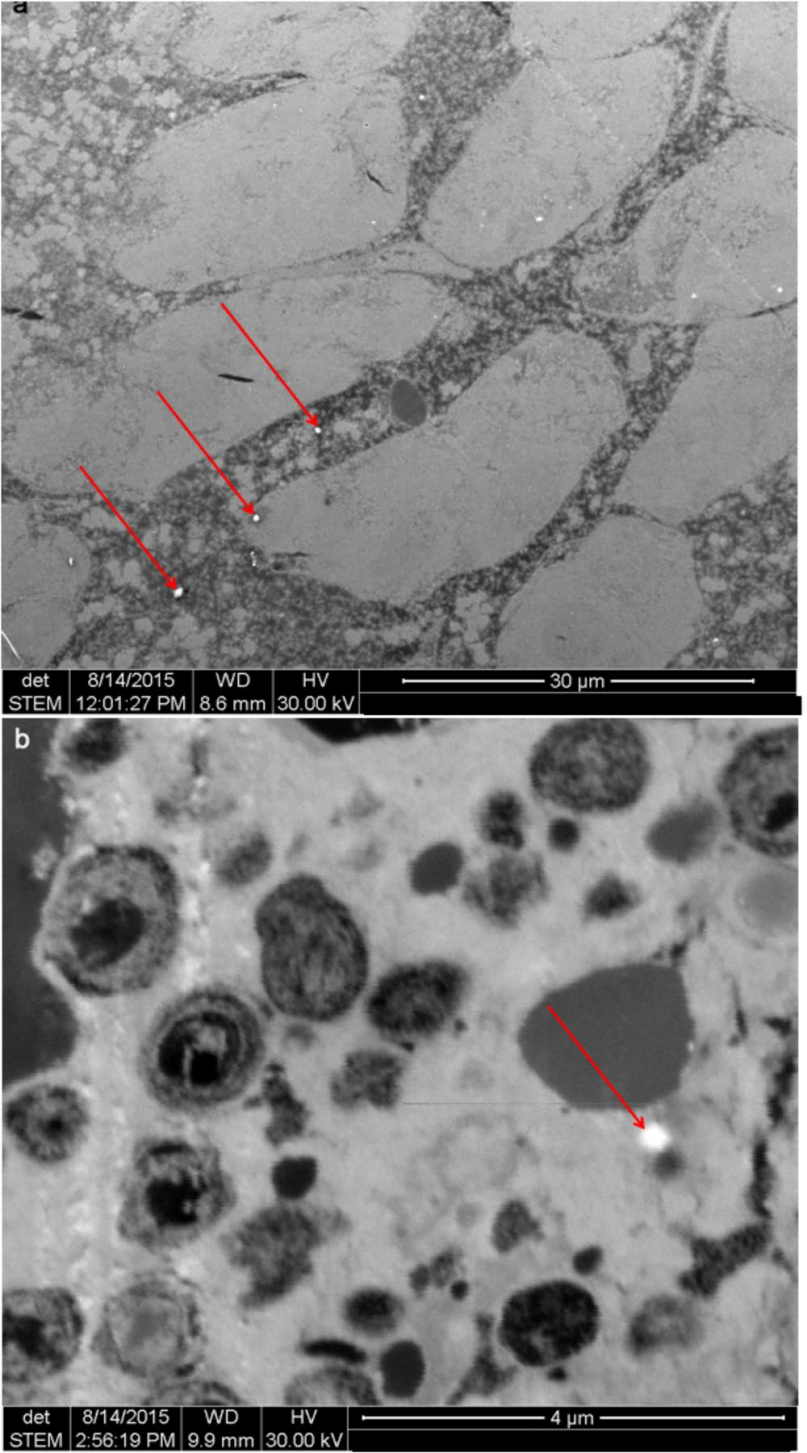
Nanoparticles in adult caddisflies. (**a**) Environmental scanning electron microscopy (ESEM) images, diagrams of the energy dispersive X-ray spectroscopy confirming that the particles are Au can be found in Fig. S2 and (**b**), ESEM image of nTiO_2_ in adult caddisflies. Arrows point to nanoparticles in the organisms’ abdominal tissue.

Most importantly, emerging aquatic insects carried substantially elevated concentrations of either TiO_2_ or Au following exposure to nanoparticles (Table 1). Environmental scanning electron microscopy coupled to energy dispersive X-ray spectroscopy images of thin image sections support the uptake of nanoparticles into abdominal tissue of the adult insects (Fig. 3). A potential adhesion of nanoparticles from the medium to the outer surface of the adults following pupation, as simulated by submersing control flies into the respective media, does not account for the measured TiO_2_ and Au concentrations in the body (Table S1). Our finding demonstrates the potential for nanoparticles to be transferred from the aquatic ecosystem into the terrestrial food web – a novel pathway for nanoparticles in the environment. In the gut of a terrestrial predator, nanoparticles may affect the digestive system^29^ and thus the physiological constitution. Moreover, we calculated theoretical intake rates based on the estimated daily consumption of approximately 6 g of insects during pregnancy and lactation of little brown bats (*Myotis lucifugus*)^30^ assuming that their Au concentration equals the median concentrations detected in adults of the present study. These extrapolations suggest a theoretical ingestion of nAu with the aquatic prey at levels that are a factor of 1000 above those considered as maximum intake for humans as a consequence of gold being used as food additives^31^. At the same time, it was indicated that dietary ingestion of gold at levels comparable to this maximum intake can cause skin rash in humans^32^. Although these insights are worrying for aquatic-terrestrial meta ecosystems^17^, predicting the implications of this exposure for terrestrial food webs remains difficult^33^, particularly as the present study followed a relatively simplistic experimental setting involving one species under relatively well controlled environmental conditions. Nonetheless, based on simplistic assumptions that (i) the concentration of nAu and nTiO_2_ is comparable among merolimnic insect species and (ii) environmental concentrations reach the levels tested here, nAu and nTiO_2_ fluxes from the aquatic to the terrestrial environment can be as high as 1.4–31.8 mg and 2.4–56.5 mg each per square meter of water surface per year, respectively. This estimate is based on the median nanoparticle concentrations measured in the present study and the total annual biomass of emerging merolimnic insects from streams as reviewed by Raitif *et al*.^34^. These data therefore may raise concerns about the consequences for higher trophic levels in riparian systems, including endangered species, associated with the transfer of contaminants such as nanoparticles from the aquatic back to the terrestrial food web. Although recent explorations of the movement and impact of contaminants, such as microplastics^10^, metals^11–14^, polychlorinated biphenyls^15^, pharmaceuticals^16^ and in the present study nanoparticles, highlight impairments in the aquatic-to-terrestrial subsidy, research addressing this question is still in its infancy warranting further studies^17^.

## Materials and Methods

### Nanoparticles

nTiO_2_ and nAu have been selected as model nanoparticles for the following reasons: Titanium dioxide is one of the most frequently applied nanoparticles worldwide used in personal care products, coatings, paints, and pigments^35,36^, entering the natural environment for instance via wastewater treatment plant effluents and landfill leachates. Gold, in contrast, has a low background concentration in environmental matrices, increasing the sensitivity of the chemical analyses and thus the possibility to quantify any potential transfer of nAu across ecosystem boundaries as part of the emerging insects.

The titanium dioxide product used in this study was purchased as a powder from Evonik (P 25; Germany) featuring an advertised primary particle size of 21 nm and crystalline composition of approximately 70% of anatase and 30% of rutile. Its advertised surface area was approximately 50 m^2^/g. The powder was transferred into a dispersant additive-free, size-homogenized, charge-stabilized suspension by stirred media milling (PML 2, Bühler AG, Switzerland^37^) at a concentration of 83.87 g nTiO_2_/L in deionized water.

Gold nanoparticles were synthesized by reducing Au^3+^ with tri-sodium citrate, essentially following the protocol published by McFarland *et al*.^38^. Briefly, a 1 mM hydrogen tetrachloraurate (HAuCl_4_*3H_2_O; Sigma-Aldrich, Germany) solution in sterile deionized water was stirred and heated until boiling. When the solution reached boiling, tri-sodium citrate (Na_3_C_6_H_5_O_7_; 38.8 mM; 15% of the volume of the diluted hydrogen tetrachloraurate solution) was quickly added. Heating and stirring were ceased after the solution turned a deep red color, resulting in a 79.95 mg nAu/L stock suspension. The particle size distributions of both stock suspensions were determined by dynamic light scattering (DLS; DelsaNano C, Beckman Coulter, Germany), and their development in the test medium (SAM-S5) over a 24 h period (the time of one water exchange) is described in Table S3.

### Test organisms

The test species *Chaetopteryx villosa* (4^th^ instar) was sampled from the Sauerbach, a near-natural stream negligibly impacted by settlement or agricultural activity (49°05′N; 7°37′E). In the laboratory, the animals were acclimatized to experimental conditions in stainless-steel artificial streams (120 × 30 × 20 cm; water volume 50 liters) filled with SAM-5S medium^39^ at 14 ± 1 °C for 14 days. Within each artificial stream microcosm, *Chaetopteryx* larvae (length greater than 10 mm) were kept in groups of 40 in stainless steel cages (70 × 14 × 20 cm) covered at the in-flow and out-flow with a 0.5 mm mesh screen. Each cage was equipped with artificial sediment comprised of 1.2 kg glass spheres of two size classes (1.0 and 0.2 kg with diameters of 105–210 µm and 800–1200 µm, respectively; Worf Glaskugeln GmbH, Germany). Glass spheres were used instead of natural sediment to minimize cross contamination with titanium from sources other than the water. Cages were deployed directly in front of the paddle wheel, which simulated running water conditions at a velocity of 0.1 m/s within the cages.

### Experimental set-up

In total, the experiment involved 24 independent stainless-steel artificial streams, while each of the six independent technical control units (Pequitec, Switzerland) supplied four streams with the respective test medium (i.e., SAM-5S; water quality parameters are detailed in Table S4). Thus, a continuous medium exchange in the experimental units was ensured (one complete water exchange per 24 h), as well as dosing with the respective nanoparticles (see below). Dosing was initiated following the 14-day acclimatization phase. The artificial stream system was coupled with a light system simulating daylight and emitting UV-A and UV-B (Magic Sun 23/160 R 160 W, Heraeus, Germany) light at intensities of 9.7 and 0.35 W/m^2^, respectively, at the water surface (measured with a RM12 radiometer; Dr. Gröbel UV-Elektronic GmbH, Germany). These intensities are less than 25% of the peak UV-intensities measured during summer in Central Europe^19^ and represent values measured on a cloudy summer day near the test species sampling site^40^. Thus, the UV-intensities employed in the present study can be considered environmentally relevant, especially during the summer period. The day/night rhythm was set at 12/12 hours. Depending on the treatment, a UV-transparent or -filtering film (LLumar^®^ UV CL-SR PS, SUNPOINT, Germany) covered the artificial stream. This film also served as a barrier to emerging insects.

The experimental system was used to establish six different treatments: (i) a control without the addition of nanoparticles or direct exposure to UV-irradiation, (ii) one treatment assessing the implication of UV-irradiation in the absence of nanoparticles, (iii) one treatment assessing for the implications of 4 µg nTiO_2_/L in the absence of UV, (iv) one treatment using the same concentration of nTiO_2_ in presence of UV, (v) one treatment investigating the combined effects of 400 µg nTiO_2_/L and UV-irradiation, and (vi) one treatment that evaluated the effect of 6.5 µg nAu/L in the absence of UV. Each of these treatments was replicated four times and assessed over the course of 140 days. During the study, caddisfly larvae received approximately 10 g (dry weight) of preconditioned black alder leaves see for details^41^ every other week, while all leaf material remaining from the former addition was removed, dried (at 60 °C for 48 h) and weighed to calculate the consumed leaf mass. Subsequently, the leaves were stored frozen for chemical analysis. At the same time, dead larvae were removed from the system. In addition, each artificial stream system was checked for emerging aquatic insects daily, which were frozen until further use.

### Lipid analysis

The lipid content of 10–15 lyophilized and weighed (to the nearest 0.01 mg) adult insects per treatment was analyzed following the method described by Van Handel^42^: Lipids were extracted in a 1:1 chloroform:methanol (v:v) solution and reacted with sulfuric acid and vanillin-phosphoric acid reagent. For quantification, absorbance at 490 nm was measured and read against a standard curve prepared from commercially available soybean oil (Sojola Soja-Öl, Vandemoortele, Germany).

### Chemical analysis

To monitor nanoparticle concentrations in the test medium, water samples were taken prior to the start and once a month during the experiment and were immediately analyzed using inductively coupled plasma quadrupole mass spectrometry ICP-MS, cp.^43^. Briefly, the ICP-MS (XSeriesII, Thermo Fisher Scientific, Germany) was equipped with a FAST autosampler (ESI, Thermo Fischer Scientific, Germany), a peek spray chamber (Thermo Fischer Scientific, Germany) and a robust Mira Mist peek nebulizer (Burgener, England). The respective elements were analyzed for the following masses: ^197^Au and ^46/47/49^Ti. Additionally, the concentration of freeze-dried adult caddisflies (including any nanoparticles potentially adsorbed to the exterior of the organisms) and adult control organisms submersed in either the 400 µg nTiO_2_/L or the 6.5 µg nAu/L treatment to control for the feasible external adhesion of nanoparticles, as well as composite samples of the leaf material offered as a food source during the experiment, were analyzed via ICP-MS. The nAu samples were analyzed after digestion with aqua regia for 96 h in darkness while the temperature was increased from 30 to 80 °C. All digested samples were diluted in 1% HCl and centrifuged (3000 rpm for 15 min) to remove indigestible residues. Finally, the supernatants were analyzed by ICP-MS as described for water samples. For nTiO_2_ samples, the device was coupled with electrothermal vaporization (ETV 4000 System, Spectral Systems, Germany) for sample introduction to overcome the poor solubility of TiO_2_^44^. Prior to vaporization, the samples were incinerated (300 °C) to lower the introduction of organics. Furthermore, the instrument was run in collision cell mode with −2.5 V and 4 mL He/H_2_ cell gas in order to avoid polyatomic interference. In addition, 25 mL Ar/O_2_ gas was applied to oxidize the introduced carbon.

### TEM analyses

Each nanoparticle stock suspension (nTiO_2_ and nAu) was diluted and transferred onto a copper grid coated with carbon by ultrasonic nebulization and investigated with the 200 keV Zeiss 922 Omega transmission electron microscope. The caddisflies, in contrast, were fixed in a mixture of 2.5% glutaraldehyde, 1.25% formaldehyde, 0.03% picric acid and 0.003% CaCl_2_ in 0.1 M cacodylatbuffer (pH 7.4) for 24 h at 4 °C. Subsequently the samples were washed three times with 0.1 M cacodylatbuffer and secondary fixed using 1% OsO_4_ in 0.1 M cacodylatbuffer for 24 h at 4 °C. Samples were dehydrated by undenatured ethyl alcohol and embedded in epoxy resin (intermediate medium: 1,2-epoxypropane). The abdomen of each fly was cut crosswise with a microtome and each slice was put on a single slot copper grid (oval hole), while leaving out any type of contrasting. The specimens were investigated with the transmission electron microscope and the Quanta 325 environmental scanning electron microscope in wet scanning transmission electron microscopy mode (dark and bright field) taking advantage of the energy dispersive X-ray spectroscopy.

### Data evaluation

Emergence over time was expressed as a percent relative to the total number of emerging insects at the end of the experiment (i.e., after 140 days). On the basis of the cumulative emergence data, non-linear regression analysis was performed to identify the time by which 50% of the *Chaetopteryx* larvae had emerged [with 95% confidence interval (CI)]. For this purpose, several 2-parameter models (generally Weibull or log-normal models) were fitted to the data using the “R” extension package “drc”^45^. The model fitting the data best was selected on the basis of Akaike’s Information Criterion (i.e., lowest score; drc-function “mselect”) and visual inspection. The time until median emergence was derived using the drc-function “ED”. Obtained values were assessed for statistically significant differences between treatments using CI-testing (drc-function “comped”)^46^. The concentrations of TiO_2_, Au and lipids in emerged *Chaetopteryx* were compared based on their median or mean and (non-)parametric CI-testing methods, as detailed in Altman *et al*.^47^. CI-testing is basically a formalized approached to assess for the overlap of variability between treatments^48^. R version 3.0.3 for Mac was used for statistics and figures^49^.

## Supplementary information

Supplementary information

## Data Availability

Data are available from the corresponding authors upon request.
