# Supplementary material for: Nanoparticles transported from aquatic to terrestrial ecosystems via emerging aquatic insects compromise subsidy quality
Source: Sci Rep. 2019 Oct 30;9:15676. doi: 10.1038/s41598-019-52096-7 (PMC6821837; doi:10.1038/s41598-019-52096-7)
Supplement: Supplementary file 1 — Supplementary information [file 41598_2019_52096_MOESM1_ESM.pdf]

Supplementary Materials for

Nanoparticles transported from aquatic to terrestrial ecosystems via emerging aquatic insects compromise subsidy quality

Mirco Bundschuh, Dominic Englert, Ricki R. Rosenfeldt, Rebecca Bundschuh, Alexander Feckler, Simon Lüderwald, Frank Seitz, Jochen P. Zubrod, Ralf Schulz
Correspondence to: bundschuh@uni-landau.de; schulz@uni-landau.de

This PDF file includes:

Figures S1 & S2
Tables S1-S4
References 26-36

Other Supplementary Materials for this manuscript include the following:

none

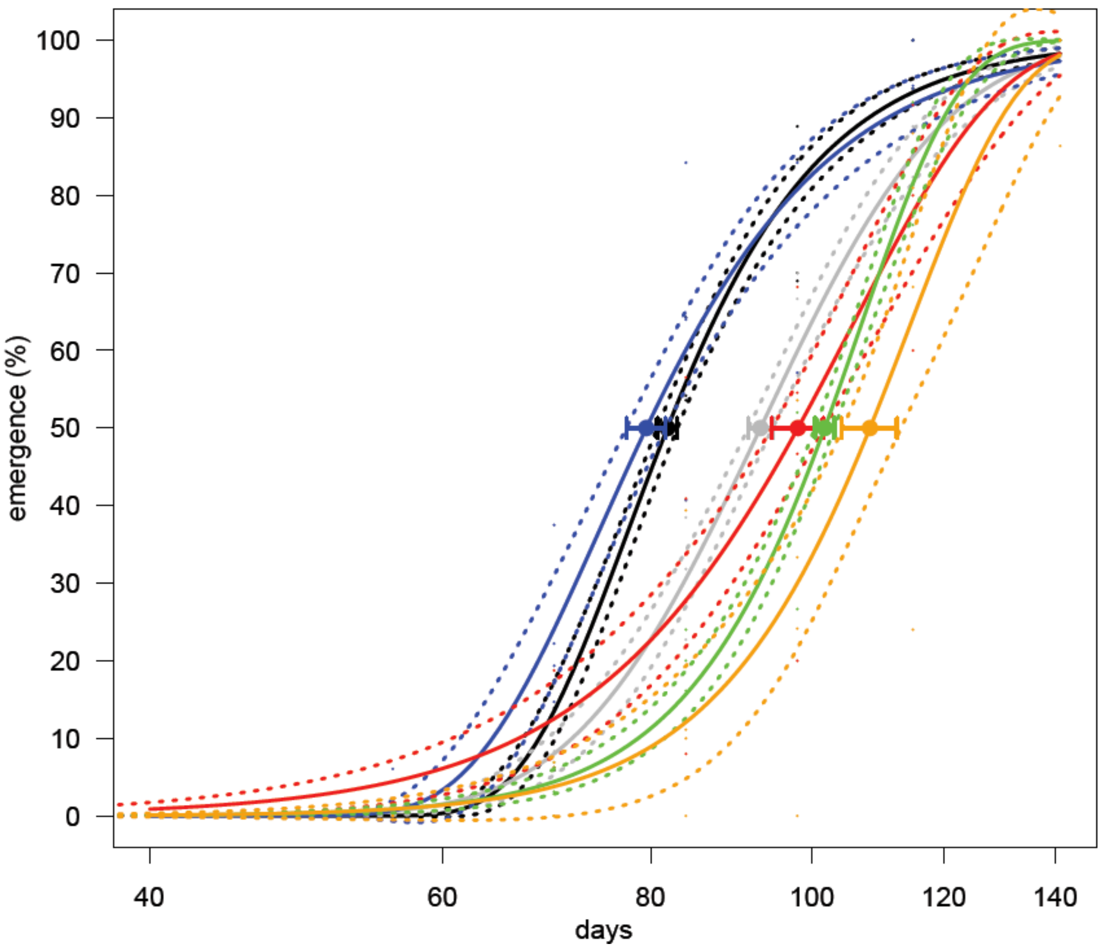

Fig. S1: **Emergence of caddisfly larvae**. The emergence (solid lines with confidence interval dashed line) of *C. villosa* under control conditions (black) and in the presence of 4 µg/L $nTiO_2$ (blue), UV-irradiation (grey), 4 µg/L $nTiO_2$ and UV-irradiation (red), 400 µg/L $nTiO_2$ and UV-irradiation (green), or nAu (orange). Bold dots represent the median time until 50% of the larvae emerged together with the respective 95% confidence interval.

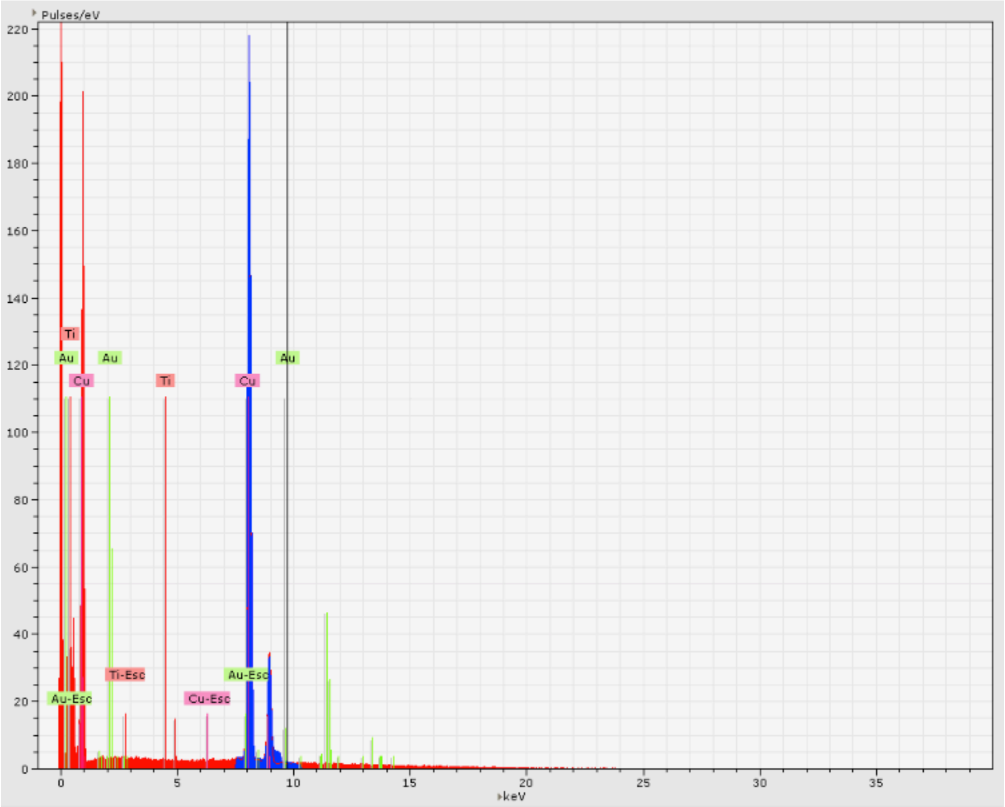

Fig. S2: **EDX-diagram confirming the presence of Au.** The EDX analysis confirmed that tissue surrounding the nanoparticles detected in Fig. 2a is containing Au suggesting that these particles are indeed nAu.

Tab. S1: Concentration of nanoparticles in the water phase, leaves and experimentally submersed adults. Mean (±standard deviation) concentration of TiO_2 and Au in the water phase (µg/L; n=9) in leaf material at the time of food renewal (i.e., after 14 days; ng/mg; n=3) under treatment conditions, and in adult *Chaetopteryx villoss* (ng/mg; n=3) emerged from control larvae but briefly submersed in medium containing either 400 µg TiO_2/L or 6.5 µg nAu/L

		TiO_2 (µg/L or ng/mg)	Au (µg/L or ng/mg)
Water phase			
	control	<LOD	<LOD
	4 µg $nTiO_2$/L	3.94 (±0.75)	NA
	400 µg $nTiO_2$/L	373.52 (±61.23)	NA
	6.5 µg nAu/L	NA	6.48 (±1.15)
Leaf material			
	control	27.30 (±3.52)	<LOD
	4 µg $nTiO_2$/L	20.69 (±11.59)	<LOD
	400 µg $nTiO_2$/L	276.00 (±128.28)	<LOD
	6.5 µg nAu/L	NA	577.34 (±55.70)
Submersed control flies			
	4 µg $nTiO_2$/L	NA	NA
	400 µg $nTiO_2$/L	0.50 (±0.64)	NA
	6.5 µg nAu/L	NA	<LOD

NA = not assessed
LOQ = absolute limit of quantification (3.6 ng for Au)

Tab. S2: Implications of nanoparticles in caddisfly emergence, survival and feeding. Mean (±standard deviation) number of emerged and dead *C. villosa* larvae together with cumulative leaf mass loss over the study duration of 140 days (n=4).

Treatment	Mean emergence (No.)	Mean death (No.)	Cumulative leaf mass loss (mg)
Control	19.0 (±6.6)	21.0 (±6.6)	773.4 (±36.5)
UV	18.8 (±4.9)	21.2 (±4.9)	754.0 (±30.7)
4 µg $nTiO_2$/L	17.8 (±4.2)	22.2 (±4.2)	710.8 (±89.9)
UV x 4 µg $nTiO_2$/L	16.3 (±4.3)	23.7 (±4.3)	716.1 (±24.5)
UV x 400 µg $nTiO_2$/L	19.5 (±3.1)	20.5 (±3.1)	738.0 (±74.0)
6.5 µg nAu/L	21.0 (±1.8)	19.0 (±1.8)	783.8 (±73.1)

Tab. S3: Nanoparticle size characteristics. Median, 10th and 90th percentiles of $nTiO_2$ and nAu size distributions at test initiation (0 h) and after 24 h in the stock suspension as well as in the test medium. In addition, the polydispersion index (PI) is reported (n=3).

Time	Nanoparticle	Medium	10th percentile (nm)	Median (nm)	90th percentile (nm)	PI
0 h	$nTiO_2$	Stock	37.4	62.3	104.1	0.1
		SAM-5S	182.8	343.2	697.5	0.2
	nAu	Stock	10.3	15.1	22.3	0.2
		SAM-5S	46.4	114.8	277.8	0.3
24 h	$nTiO_2$	Stock	37.2	61.0	100.5	0.1
		SAM-5S	392.6	1583.5	8280.9	0.6
	nAu	Stock	9.8	16.3	27.8	0.2
		SAM-5S	280.3	1412.0	29436.0	0.7

Tab. S4: Water quality parameters. Mean (±standard deviation) of water quality parameters over the duration of the study, measured weekly in one randomly selected control and any treatment (n=14; TOC n=4).

Water quality parameter	Control	Treatment
Ammonium (mg/L)	<0.02	<0.02
Phosphate (mg/L)	0.19 (±0.28)	0.28 (±0.80)
Nitrite (µg/L)	7.58 (±8.01)	6.34 (±6.07)
Nitrate (mg/L)	<1.00	<1.00
Chloride (mg/L)	35.07 (±23.63)	35.07 (±23.63)
Oxygen (mg/L)	9.36 (±1.21)	9.61 (±1.29)
Temperature (°C)	14.07 (±0.19)	13.94 (±0.30)
pH	8.22 (±0.25)	8.18 (±0.26)
Conductivity (µS/cm)	361 (±139)	378 (±116)
Hardness (°d)	7.86 (±0.58)	7.86 (±0.49)
Total organic carbon (mg/L)	11.54 (±0.68)	10.12 (±1.13)
